# Avoidable Mortality in Adults With Down Syndrome or With Other Intellectual Disabilities

**DOI:** 10.1001/jamanetworkopen.2026.43229

**Published:** 2026-11-11

**Authors:** Lili Bokor, Michael Kwan Leung Yu, Ben Carter, Umesh Chauhan, Rory Sheehan, André Strydom

**Affiliations:** 1 Department of Forensic and Neurodevelopmental Sciences, Institute of Psychiatry, Psychology and Neuroscience, King’s College London, United Kingdom; 2 Department of Biostatistics & Health Informatics, Institute of Psychiatry, Psychology and Neuroscience, King’s College London, United Kingdom; 3 School of Medicine and Dentistry, University of Lancashire, Preston, United Kingdom; 4 Oxleas National Health Service Foundation Trust, London, United Kingdom; 5 South London and Maudsley National Health Service Foundation Trust, London, United Kingdom

## Abstract

**Importance:**

Down syndrome is the most common genetic cause of Alzheimer disease. Adults with Down syndrome experience higher mortality than the general population. However, the avoidable causes and their associated factors remain unclear.

**Objective:**

To compare the proportion and causes of avoidable mortality in adults with Down syndrome, adults with other intellectual disabilities, and the general population, and to identify factors associated with avoidable death.

**Design, Setting, and Participants:**

This population-based retrospective cohort study used data from the Learning From Lives and Deaths mortality review and the Office for National Statistics in England from 2016 to 2023. It included adults with Down syndrome (n = 3381), other adults with intellectual disabilities (n = 15 347), and the general population (n = 4 163 160).

**Exposures:**

Down syndrome status, ethnicity, socioeconomic deprivation, co-occurring health conditions, COVID-19 pandemic period.

**Main outcomes and measures:**

Proportion and causes of avoidable, treatable, and preventable deaths according to Organisation for Economic Co-operation and Development/Eurostat criteria; odds of avoidable death estimated using logistic regression; and age at avoidable death using Cox regression. Factors associated with avoidable death and age at death were also evaluated.

**Results:**

Among 3381 adults with Down syndrome (1892 male [56.0%]; median [IQR] age at death, 59.5 [54.3-64.2] years), 1589 (47.0%) died from avoidable causes. Among 15 347 adults with other intellectual disabilities (8675 male [56.5%]; median [IQR] age at death, 63.8 [51.7-73.4] years), 6885 (44.9%) died from avoidable causes. Both proportions were substantially higher than in the general population (22.0%) (both *P* < .001). Treatable deaths were more common (972 [28.7%] vs 3847 [25.1%] vs 317 968 [7.6%]), with epilepsy and pneumonia as the leading treatable causes. Down syndrome was independently associated with higher odds of avoidable death (aOR, 1.15; 95% CI, 1.06-1.25) and earlier age at avoidable death (aHR, 1.49; 95% CI, 1.40-1.58). Minoritized ethnic background, asthma, diabetes, and osteoporosis were each independently associated with higher odds of avoidable death, and minoritized ethnic background and greater socioeconomic deprivation with earlier age at avoidable death.

**Conclusions and relevance:**

In this cohort study of adults with Down syndrome and other adults with intellectual disabilities in England, avoidable mortality exceeded that of the general population. For the adults with Down syndrome, epilepsy and pneumonia were the dominant treatable causes. These findings support proactive integrated neurological care, including epilepsy review with dementia monitoring, pneumonia prevention, and respiratory vaccination, alongside targeted interventions for minoritized ethnic groups and socioeconomically deprived groups.

## Key Points

**Question:**

Do adults with Down syndrome (DS) or with other intellectual disabilities (ID) experience a disproportionate burden of avoidable mortality, and if so, which factors are associated with it?

**Findings:**

In a cohort study of 3381 adults with DS and 15 347 adults with other ID who died in England, 47.0% and 44.9% of deaths, respectively, were avoidable, both higher than in the general population (22.0%). Epilepsy and pneumonia were the dominant treatable causes; minoritized ethnic groups, socioeconomic deprivation, asthma, diabetes, and osteoporosis were each independently associated with avoidable death.

**Meaning:**

These findings suggest adults with DS and other adults with ID represent a high-risk group requiring systematic epilepsy review, proactive pneumonia prevention, respiratory vaccination prioritization, and targeted care for minoritized ethnic groups and socioeconomically deprived groups.

## Teaser

This cohort study investigates the proportion and causes of avoidable mortality in adults with Down syndrome compared with those with other intellectual disabilities and the general population.

## Introduction

Down syndrome is the most common genetic cause of intellectual disability, affecting approximately 1 in 800 births worldwide.^1^ Despite advances in cardiac care and the management of comorbidities,^2,3^ adults with Down syndrome continue to experience a higher all-cause mortality than both the general population and adults with intellectual disabilities without Down syndrome,^4,5^ including a substantial proportion of avoidable deaths, defined as deaths before age 75 years that could potentially be averted through effective public health interventions (preventable mortality) or through timely and appropriate health care (treatable mortality).^6^

Previous research has shown that adults with Down syndrome have distinct comorbidity profiles.^7,8,9,10,11,12,13^ For example, the incidence of dementia was nearly 95-fold higher (incidence rate ratio [IRR], 94.7; 95% CI, 69.9-128.4) and epilepsy was nearly 10-fold higher (IRR, 9.7; 95% CI, 8.5-10.9) after adjustment for age, sex, and calendar year compared with general population controls.^7^ Another study confirmed that pneumonia incidence was 4.7- to 5.7-fold higher in people with Down syndrome than in matched controls and that 1-year mortality after pneumonia is significantly elevated (5.7% vs 2.4%).^9^

However, critical gaps remain in understanding avoidable mortality in adults with Down syndrome. Studies of avoidable mortality in people with intellectual disabilities have generally considered this population as a whole,^4,5^ providing limited insight into whether adults with Down syndrome have a different avoidable mortality profile. It also remains unclear whether sociodemographic factors, such as ethnicity, socioeconomic deprivation, and common comorbidities are associated with avoidable death or earlier age at death from avoidable causes. Addressing these gaps is essential for developing targeted public health strategies and clinical interventions for this population. The current study aimed to describe and compare the proportion of avoidable deaths and their underlying causes in adults with Down syndrome with those in other adults with intellectual disabilities and the general adult population and identify sociodemographic and clinical factors associated with avoidable death and age at avoidable death in adults with Down syndrome.

## Methods

The English national mortality review for people with intellectual disabilities and autistic people—the Learning From Lives and Deaths (LeDeR) program—holds Section 251 approval from the UK Health Research Authority’s Confidentiality Advisory Group, which granted the waiver of ethics committee approval for the processing of identifiable data without consent (Reference: 20/CAG/0067). This study followed the Strengthening the Reporting of Observational Studies in Epidemiology (STROBE) reporting guideline for cohort studies.

### Data Sources

The LeDeR program is an internationally unique dataset compiled to address health inequalities and reduce preventable mortality among people with intellectual disability and autistic people. Any death of a person with intellectual disabilities or autism aged 18 years or over can be notified to LeDeR. The notification is assigned to a trained reviewer who accesses the person’s medical records and speaks to their personal or professional contacts. Reviews captured individual-level sociodemographic characteristics and clinical information, including the Medical Certificate of Cause of Death (MCCD). From 2021 onwards, LeDeR reviews incorporated structured coded data to record the presence of co-occurring physical and mental health conditions at the time of death.

To compare the proportion of avoidable deaths and their underlying causes, we also extracted aggregate mortality data for the general population in England from the UK Office for National Statistics (ONS), comprising counts of deaths by underlying cause, sex, and age group.^14^ We also extracted anonymized individual-level age-at-death data from the ONS to compare survival patterns between adults with intellectual disabilities and the general population. ONS is the national provider of official demographic and health statistics in the United Kingdom.

### Study Population

Adults with intellectual disabilities with Down syndrome who died from any cause and whose deaths were reported to LeDeR between 2016 and 2023 were included in this study. Two comparison groups were included: adults with intellectual disabilities without Down syndrome from the LeDeR dataset, and adults aged at least 20 years from the general population identified from ONS mortality statistics.

### Variables

Mortality data were classified according to the presence or absence of Down syndrome using coded variables and open text fields from review data, and cause of death recorded on the MCCD (*International Statistical Classification of Diseases and Related Health Problems, Tenth Revision *[*ICD-10*] codes: Q90.0, Q90.1, Q90.2, and Q90.9). Sociodemographic variables included sex, ethnicity, index of multiple deprivation (IMD), place of death, region, and age at death. Ethnicity was recorded by LeDeR reviewers using the standard ethnicity classification derived from the ONS,^15^ categorized as: Asian or Asian British; Black, African, Caribbean or Black British; mixed or multiple ethnic groups; White; other ethnic groups; and prefer not to say or not known. Other ethnic groups included Arab and any other ethnic group not listed previously, based on ONS classification. Ethnicity was included because reducing health inequalities is one of the core aims of the study. IMD was derived from home postcode data, where 1 represents the most deprived areas and 10 the least deprived, to represent socioeconomic deprivation. Co-occurring physical and mental health conditions were derived from coded data. Underlying cause of death was gathered from the MCCD. When Down syndrome was recorded as the underlying cause of death, we used the next condition listed in Part I of the MCCD as the cause of death for the purposes of the analysis. Avoidable deaths were defined using the Organisation for Economic Co-operation and Development/Eurostat criteria (eTable 1 in Supplement 1).^6^ Within this framework, avoidable deaths comprise those classified as preventable, treatable, or both, with deaths from causes appearing on both lists apportioned equally between the 2 categories. Preventable causes are those avoidable through primary prevention, such as lung cancer (*ICD-10*, C33-C34), whereas treatable causes are those avoidable through timely and effective health care, such as epilepsy (*ICD-10,* G40, G41). The classification applies only to deaths occurring before the age of 75 years. The COVID-19 pandemic period was defined according to the World Health Organization Public Health Emergency of International Concern and modeled as a 3-level variable: prepandemic (before January 30, 2020), pandemic (January 30, 2020 to May 5, 2023), and postpandemic (after May 5, 2023).

### Statistical Analysis

We used χ^2^ tests to compare the most common and avoidable causes of death between 3 groups. Kaplan-Meier survival curves were used to compare age at death across the three groups, between avoidable and nonavoidable deaths within the Down syndrome group, and by Down syndrome status and level of intellectual disability.

To examine factors associated with avoidable death, log-binomial and logistic regression models were fitted to estimate risk ratios (RR) and odds ratios (OR), respectively, with 95% CIs among all adults with intellectual disabilities, adjusted for sex, ethnicity, socioeconomic deprivation (measured by IMD), COVID-19 period, and Down syndrome status. To examine factors associated with earlier age at avoidable death, Cox proportional hazards regression was fitted to estimate hazard ratios (HR) with 95% CIs among all adults with intellectual disabilities with avoidable deaths, with the same covariates. Subgroup analyses were conducted among all adults with Down syndrome and those with available coded data on co-occurring conditions. Sensitivity analyses were conducted among those who died before COVID-19 and during the COVID-19 period. To account for the small numbers in individual categories, ethnicities other than White (Asian or Asian British; Black, African, Caribbean, or Black British; mixed or multiple ethnic groups, or other ethnic groups) were grouped into a single category in the models. Cases were excluded from the regression models when information on sex, ethnicity, or IMD was missing or not known.

Data analyses and graphing were performed using SPSS version 32.0 (IBM Corp), R version 4.4.1 (R Project for Statistical Computing), and GraphPad Prism version 11.0.0 (GraphPad). Statistical significance was defined as a 2-sided *P* value <.05. No adjustment was made for multiple comparisons.

## Results

### Sociodemographic Characteristics and Co-Occurring Conditions

This cohort study included 3381 adults with Down syndrome (1892 male [56.0%]; median age at death, 59.5 years; IQR, 54.3-64.2), 15 347 adults with intellectual disabilities without Down syndrome (8675 male [56.5%]; median age at death, 63.8 years; IQR, 51.7-73.4), and 4 163 160 adults from the general population (2 083 085 male [50.0%]; median age at death, 81.9 years; IQR, 72.3-89.1) (Table 1; eFigure 1 in Supplement 1). Among adults with Down syndrome, 62 (1.8%) were Asian or Asian British, 56 (1.7%) were Black, African, Caribbean, or Black British, 20 (0.6%) were of mixed or multiple ethnic groups, 2749 (81.3%) were White, and 15 (0.4%) were of other ethnic groups; ethnicity was not known or not stated for 479 (14.2%). Overall, 751 (22.2%) resided in the most deprived areas (Table 1). The most common co-occurring conditions at the time of death were dementia (63.0% [742 of 1177]), dysphagia (60.5% [712]), and visual impairment (36.4% [428]) (eTable 2 in Supplement 1).

**Table 1.** Sociodemographic Characteristics of Participants by Presence of Down Syndrome and General Adult Population Comparison

Characteristic	Decedents, No. (%)		
	Group 1: adults with ID with DS (n = 3381)	Group 2: adults with ID without DS (n = 15 347)	Group 3: general adult population (n = 4 163 160)^a^
Sex			
Female	1476 (43.7)	6597 (43.0)	2 080 075 (50.0)
Male	1892 (56.0)	8675 (56.5)	2 083 085 (50.0)
Not known	13 (0.4)	75 (0.5)	NA
Ethnicity			
Asian or Asian British	62 (1.8)	489 (3.2)	NA
Black, African, Caribbean, or Black British	56 (1.7)	247 (1.6)	NA
Mixed or multiple ethnic groups^b^	20 (0.6)	104 (0.7)	NA
White	2749 (81.3)	12 492 (81.4)	NA
Other ethnic groups^c^	15 (0.4)	135 (0.9)	NA
Preferred not to say or not known	479 (14.2)	1880 (12.2)	NA
Index of multiple deprivation			
1-2 (Most deprived)	751 (22.2)	4124 (26.9)	857 299 (20.6)
3-4	809 (23.9)	3475 (22.6)	830 708 (20.0)
5-6	720 (21.3)	3113 (20.3)	856 274 (20.6)
7-8	617 (18.2)	2576 (16.8)	840 674 (20.2)
9-10 (Least deprived)	430 (12.7)	1792 (11.7)	778 205 (18.7)
Unknown	54 (1.6)	267 (1.7)	NA
Place of death			
Hospital	1782 (52.7)	9236 (60.2)	NA
Care homes	366 (10.8)	1233 (8.0)	NA
Private homes	1097 (32.4)	3988 (26.0)	NA
Hospices	48 (1.4)	335 (2.2)	NA
Elsewhere	40 (1.2)	348 (2.3)	NA
Not known	48 (1.4)	207 (1.3)	NA
Region of England			
East of England	169 (5.0)	801 (5.2)	478 151 (11.5)
London	371 (11.0)	1653 (10.8)	412 924 (9.9)
Midlands	870 (25.7)	3959 (25.8)	843 073 (20.3)
North East and Yorkshire	576 (17.0)	2608 (17.0)	671 256 (16.1)
North West	460 (13.6)	2104 (13.7)	602 902 (14.5)
South East	525 (15.5)	2330 (15.2)	680 922 (16.4)
South West	362 (10.7)	1480 (9.6)	473 932 (11.4)
Not known	48 (1.4)	412 (2.7)	NA
Age at death, y			
18-24	33 (1.0)	649 (4.2)	9784 (0.2)
25-49	433 (12.8)	2814 (18.3)	164 945 (4.0)
50-64	2193 (64.9)	4685 (30.5)	440 081 (10.6)
≥65	722 (21.4)	7199 (46.9)	3 548 350 (85.2)

Abbreviations: DS, Down syndrome; ID, intellectual disability; NA, not applicable.

^a^ Data on deaths by ethnic group, place of death, and region of England were not available from the Office for National Statistics (ONS) for these age groups. The only general adult population data available from the ONS relate to those aged 20 years and over.

^b^ Mixed or multiple ethnic groups included White and Asian, White and Black African, White and Black Caribbean and other mixed or multiple ethnic groups, based on ONS classification.^15^

^c^ Other ethnic groups included Arab and any other ethnic group not listed previously, based on ONS classification.^15^

### Age at Death

Median age at death was lower among adults with Down syndrome (59.5 years [IQR, 54.3-64.2]) compared with adults with intellectual disabilities without Down syndrome (63.8 years [IQR, 51.7-73.4]) and the general population (81.9 years [IQR, 72.3-89.1]) (*P* < .001) (Figure 1A). Within the Down syndrome group, deaths from avoidable causes occurred at a younger median age than nonavoidable deaths (58.6 vs 60.2 years; *P* < .001) (Figure 1B). Median age at death did not differ by intellectual disability severity within the Down syndrome group (*P* = .22), unlike other adults with intellectual disabilities (eFigure 2 in Supplement 1).

**Figure 1.**
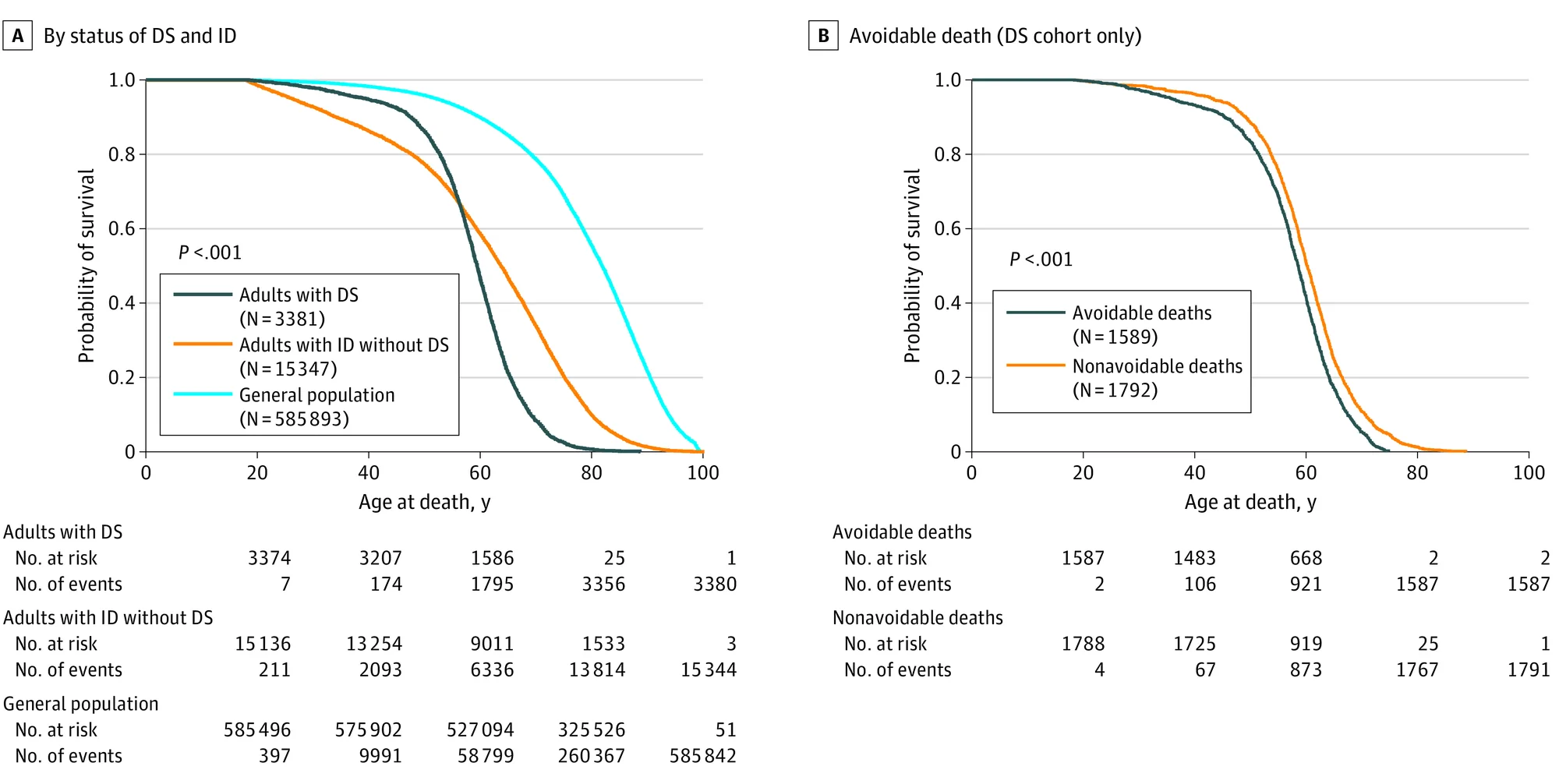
Survival Curves General population data: adults in England and Wales in 2022 (Office for National Statistics; n = 585 893). DS indicates Down syndrome; ID, intellectual disability.

### Avoidable Mortality

The proportion of avoidable deaths in adults with Down syndrome (47.0% [1589 of 3381]) and other adults with intellectual disabilities (44.9% [6885 of 15 347]) was substantially higher than in the general population (22.0% [914 866 of 4 163 160]) (both *P* < .001) (Table 2). Treatable deaths accounted for 28.7% (972) of deaths in adults with Down syndrome, 3847 of 15 347 (25.1%) in other adults with intellectual disabilities, and 317 968 of 4 163 160 (7.6%) in the general population (both *P* < .001). Epilepsy (8.6% [290]) and pneumonia (6.0% [203]) were the leading treatable causes in adults with Down syndrome; epilepsy-related deaths were more frequent than in both comparison groups (both *P* < .001) while pneumonia-related deaths were higher than in the general population (*P* < .001).

**Table 2.** Percentage and Leading Causes of Avoidable Mortality by Down Syndrome Status, 2016 to 2023

Cause or category of avoidable mortality	Decedents, No. (%)			*P* value	
	Group 1: adults with ID with DS (n = 3381)	Group 2: adults with ID without DS (n = 15 347)	Group 3: general adult population (n = 4 163 160)	Group 1 vs group 2	Group 1 vs group 3
Category of avoidable mortality					
Avoidable	1589 (47.0)	6885 (44.9)^a^	914 866 (22.0)	.02	<.001
Treatable	972 (28.7)	3847 (25.1)	317 968 (7.6)	<.001	<.001
Preventable	617 (18.2)	3038 (19.8)	596 898 (14.3)	.04	<.001
Most common causes of treatable mortality					
Epilepsy	290 (8.6)	470 (3.1)	4357 (0.1)	<.001	<.001
Pneumonia, not elsewhere classified or organism unspecified	203 (6.0)	980 (6.4)	26 883 (0.6)	.41	<.001
Congenital malformations of the circulatory system (heart defects)	79 (2.3)	67 (0.4)	2295 (0.1)	<.001	<.001
Acute lower respiratory infections	42 (1.2)	153 (1.0)	31 846 (0.8)	.20	.001
Venous thromboembolism	40 (1.2)	214 (1.4)	17 279 (0.4)	.34	<.001
Most common causes of preventable mortality					
COVID-19	336 (9.9)	970 (6.3)	44 804 (1.1)	<.001	<.001
Lung diseases due to external agents	65 (1.9)	271 (1.8)	2898 (0.1)	.53	<.001
Accidental injuries	22 (0.7)	174 (1.1)	78 631 (1.9)	.01	<.001
Influenza	17 (0.5)	51 (0.3)	6864 (0.2)	.14	<.001
Chronic lower respiratory diseases	13 (0.4)	216 (1.4)	203 139 (4.9)	<.001	<.001
Most common both treatable and preventable mortality					
Cerebrovascular diseases	121 (3.6)	470 (3.1)	48 926 (1.2)	.12	<.001
Ischemic heart diseases	64 (1.9)	650 (4.2)	152 335 (3.7)	<.001	<.001
Diabetes	44 (1.3)	106 (0.7)	52 004 (1.2)	<.001	.79
Hypertensive diseases	6 (0.2)	90 (0.6)	57 832 (1.4)	.003	<.001
Aortic aneurysm	NA^b^	24 (0.2)	37 271 (0.9)	.24	<.001

Abbreviations: DS, Down syndrome; ID, intellectual disability; NA, not applicable.

^a^ *P* < .001 for the comparison between Group 2 and Group 3.

^b^ Suppressed for those with fewer than 5 cases, instead of those that were imputed as 3 cases for statistical test.

Preventable deaths accounted for 18.2% (617) of deaths, lower than in other adults with intellectual disabilities (19.8% [3038 of 15 347]; *P* = .04) but higher than in the general population (14.3% [596 898 of 4 163 160]) (*P* < .001). COVID-19 was the leading preventable cause of death (9.9% [336]) and was more common than in both comparison groups (both *P* < .001). Age-specific patterns of avoidable mortality are presented in eTable 3 in Supplement 1.

### Factors Associated With Avoidable Deaths

Among all adults with intellectual disabilities, Down syndrome was independently associated with higher odds of avoidable death (adjusted odds ratio [aOR], 1.15; 95% CI, 1.06-1.25; *P* < .001) (Figure 2A). Within the Down syndrome cohort, minoritized ethnic background (aOR, 1.56; 95% CI, 1.11-2.18; *P* = .01) and death during the COVID-19 period (aOR, 1.39; 95% CI, 1.18-1.63; *P* < .001) were associated with higher odds of avoidable death.

**Figure 2.**
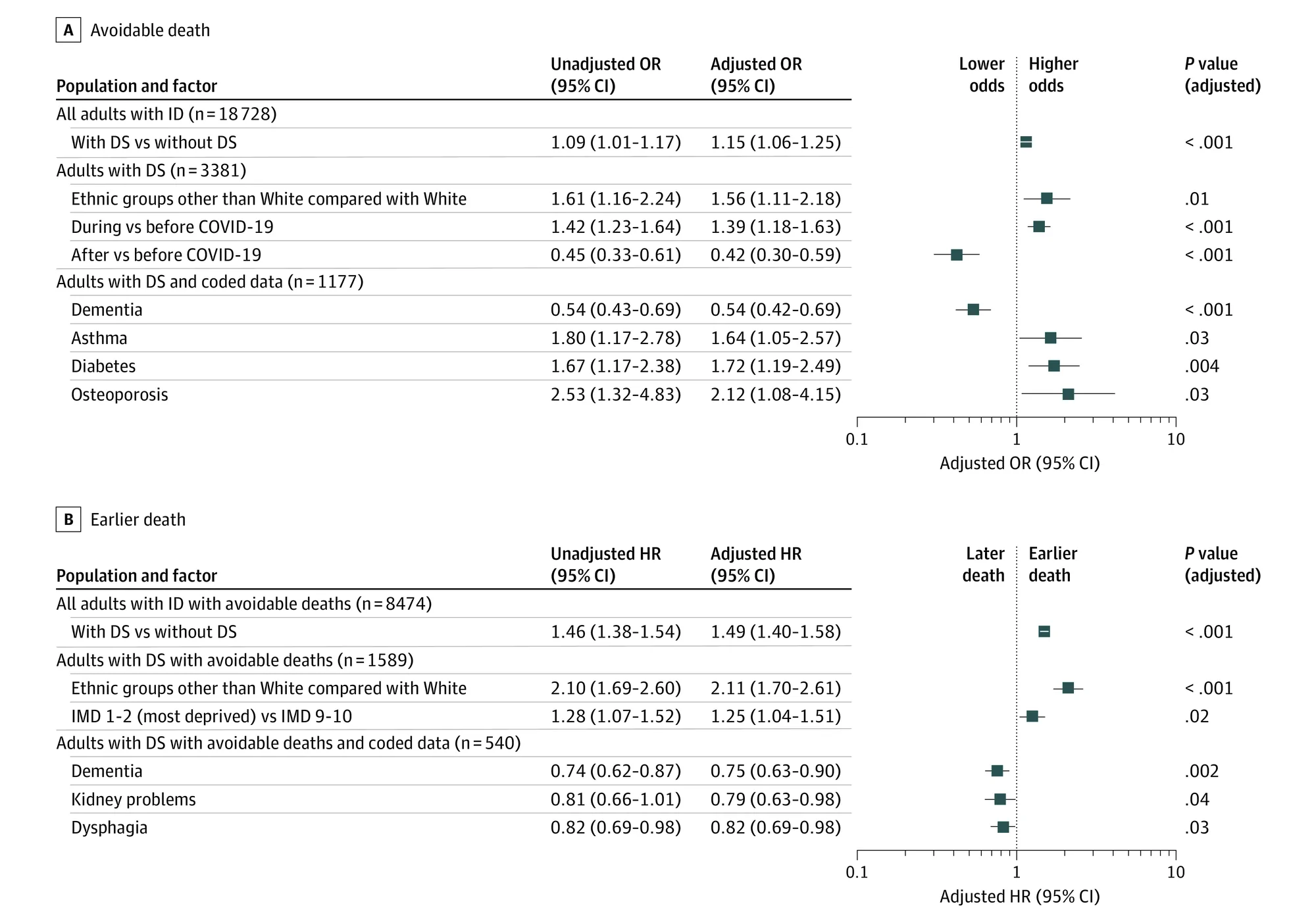
Dot Plot of Factors Associated With Avoidable Death in Adults With Down Syndrome DS indicates Down syndrome; HR, hazard ratio; ID, intellectual disability; OR, odds ratio.

In analyses restricted to individuals with coded comorbidity data, osteoporosis (aOR, 2.12; 95% CI, 1.08-4.15; *P* = .03), diabetes (aOR, 1.72; 95% CI, 1.19-2.49; *P* = .004), and asthma (aOR, 1.64; 95% CI, 1.05-2.57; *P* = .03) were associated with higher odds of avoidable death, whereas dementia was associated with lower odds (aOR, 0.54; 95% CI, 0.42-0.69; *P* < .001) (eTable 4 in Supplement 1). Log-binomial model results are shown in eTable 5 in Supplement 1. Down syndrome was again independently associated with a higher risk of avoidable death (adjusted risk ratio [aRR], 1.08; 95% CI, 1.04-1.13; *P* < .001), and the other associations were consistent in direction and significance with the logistic regression estimates.

### Factors Associated With Age at Death Among Adults With Avoidable Deaths

Among adults with intellectual disabilities who died from avoidable causes, Down syndrome was associated with earlier age at death (adjusted hazard ratio [aHR], 1.49; 95% CI, 1.40-1.58; *P* < .001) (Figure 2B). Within the Down syndrome cohort, status as a minoritized ethnic group (aHR, 2.11; 95% CI, 1.70-2.61; *P* < .001) and residing in the most deprived areas (aHR, 1.25; 95% CI, 1.04-1.51; *P* = .02) were associated with earlier death.

In subgroup analyses with coded comorbidity data, dementia (aHR, 0.75; 95% CI, 0.63-0.90; *P* = .002), kidney problems (aHR, 0.79; 95% CI, 0.63-0.98; *P* = .04), and dysphagia (aHR, 0.82; 95% CI, 0.69-0.98; *P* = .03) were associated with later age at death. When the analysis was restricted to those with dementia, the associations of kidney problems and dysphagia with age at death were no longer statistically significant (eTable 6 in Supplement 1).

Full regression results, including the unadjusted models and sensitivity analyses by COVID-19 period are presented in eTables 4 to 10 in Supplement 1. Estimates differed between deaths occurring before and during the COVID-19 pandemic, suggesting that associations may vary by pandemic period.

## Discussion

This population-based cohort study found that nearly half of deaths among adults with Down syndrome were classified as avoidable, higher than the general population. This excess was largely related to treatable deaths, particularly due to epilepsy and pneumonia, while COVID-19 was the leading preventable cause of avoidable deaths. Ethnic background other than White, the COVID-19 period, asthma, diabetes, and osteoporosis were independently associated with higher odds of avoidable death. Among those who died from avoidable causes, individuals with a minoritized ethnic background and greater socioeconomic deprivation were also associated with earlier age at death. Notably, survival did not differ by level of intellectual disability among adults with Down syndrome, an observation that contrasts with patterns seen in other adults with intellectual disabilities. However, adults with intellectual disabilities without Down syndrome also experienced a higher overall burden of avoidable mortality than the general population, underscoring that excess avoidable mortality extends to the wider intellectual disability population. As this comparison group includes a heterogeneous range of conditions, additional research is needed to characterize avoidable mortality within specific subgroups.

The results add to findings from smaller existing studies. A Scottish cohort study of 57 adults with Down syndrome reported that 29.8% of deaths were classified as avoidable,^5^ while a Danish nationwide cohort study found that adults with intellectual disabilities, including those with Down syndrome, had a 6-fold higher rate of preventable mortality and a 2.62-fold higher rate of treatable mortality than an age- and sex-matched reference cohort.^16^ Our data show that treatable deaths accounted for approximately two-thirds of avoidable mortality in adults with Down syndrome, with epilepsy and pneumonia as major contributors. The disproportionate burden of epilepsy-related deaths likely reflects the neurobiological intersection of trisomy 21 and Alzheimer disease pathology. Amyloid-driven cortical hyperexcitability in Alzheimer disease gives rise to epileptiform activity and seizures, which may both accelerate cognitive decline and increase mortality risk.^17,18^ These findings support calls for integrated epilepsy management, including proactive dementia monitoring and regular antiseizure medication review tailored to the Alzheimer disease trajectory in this population.^7,19,20^ In the context of immunological abnormalities associated with trisomy 21,^9^ adults with Down syndrome may also benefit from more proactive pneumonia prevention and prompt clinical intervention, including timely diagnosis, severity assessment, prompt antibiotic treatment, and prevention strategies in people at higher risk of severe disease.^21^ Furthermore, the elevated burden of pneumonia-associated deaths also likely reflects aspiration risk conferred by dysphagia,^8^ which is common in this group.^22^

Preventable COVID-19 deaths were more common among adults with Down syndrome. This finding is consistent with evidence that people with Down syndrome had elevated COVID-19 mortality risk.^23,24^ The proportion of influenza deaths was also higher in adults with Down syndrome than in the general population, consistent with findings from a US study.^25^ These data lend weight to the prioritization of adults with Down syndrome for respiratory vaccination, with accessible communication and reasonable adjustments to improve uptake.^26,27,28^

This study is the first to examine the sociodemographic and clinical factors associated with avoidable death and age at avoidable death among adults with Down syndrome. Minoritized ethnic background was independently associated with both higher odds of avoidable death and earlier age at death, consistent with wider evidence.^29^ Adults in the most deprived areas also faced earlier avoidable death, consistent with Norwegian registry findings.^30^ These findings suggest that minoritized ethnic groups and people living in deprived areas may face additional barriers to accessing timely health care.^31^

The COVID-19 period was associated with increased odds of avoidable death, suggesting that the pandemic had marked adverse associations with avoidable mortality in this population, consistent with evidence that people with Down syndrome were at particularly high risk of severe COVID-19 outcomes and death.^23,24,32^ The COVID-19 pandemic period was also associated with widespread disruption to health care services, which may have contributed to non-COVID-19 avoidable deaths.^33,34,35,36,37^

Asthma, diabetes, and osteoporosis were associated with higher odds of avoidable death. These conditions may indicate greater clinical vulnerability in adults with Down syndrome, for example, asthma and diabetes mellitus both are associated with increased risk of pneumococcal disease and respiratory infection, including severe COVID-19,^38,39^ which were among the most common preventable causes of death in this study. In addition, osteoporosis has been associated with increased risk of falls and fractures,^40^ which may contribute to preventable mortality due to accidental injuries. These findings suggest that adults with Down syndrome who have asthma, diabetes, or osteoporosis may benefit from more targeted health care, including strategies to prevent infection and falls.

In contrast, dementia, kidney problems, and dysphagia were associated with later age at death among those with avoidable deaths. These findings should be interpreted cautiously, however, as previous studies have generally linked dementia in adults with Down syndrome to increased overall mortality.^41,42^ As these conditions are age-related, associations^43,44^ with later age at death may reflect healthy survivor effects rather than true protective effects.

### Limitations

This study has several limitations. First, there is potential for sampling bias. Although reporting a death to the LeDeR program is encouraged, notification is not mandatory; therefore, some deaths in England may have gone unreported. As a result, mortality ratios could not be estimated in comparison with the reference groups. Furthermore, under ascertainment may not be uniform and could affect the associations we observed with ethnicity, socioeconomic deprivation, and region. Because the direction of any differential notification cannot be determined, these estimates could be biased either upward or downward. Second, the LeDeR dataset is limited to mortality records and does not provide information on the age distribution of the living population with intellectual disabilities. This restricts our ability to assess age-related trends within subgroups, particularly among minoritized ethnic groups and for co-occurring conditions. Therefore, findings related to age at death by ethnicity should be interpreted with caution; if surviving adults with intellectual disabilities from minoritized ethnic backgrounds are younger on average than their White counterparts, this may have influenced the results of regression models. Third, completeness of data on co-occurring conditions was limited, with coded information on co-occurring conditions only available for reviews completed between 2021 and 2024, which may reduce the generalizability of conclusions regarding condition prevalence and patterns. In addition, obesity was not recorded in the coded dataset. Fourth, the aggregated ONS general population mortality dataset used as a comparator includes deaths among people with Down syndrome and intellectual disabilities,^45^ which may have slightly attenuated the observed differences in causes of death between populations.

## Conclusions

This population-based cohort study found that adults with Down syndrome in England had a higher proportion of avoidable deaths than both the general population and other adults with intellectual disabilities. Nearly half of all deaths were avoidable and associated predominantly with treatable causes, epilepsy and pneumonia being foremost among these. COVID-19 was the leading preventable cause, and minoritized ethnic group background and socioeconomic deprivation compounded mortality risk. These findings identify adults with Down syndrome as a high-priority subgroup requiring integrated, proactive care, including structured epilepsy review linked to dementia monitoring, respiratory infection vaccination and early treatment, and targeted outreach to those facing the greatest health inequities. Many of these priorities also apply across the wider intellectual disability population. Future research should evaluate how to deliver and target interventions that can reduce avoidable deaths from epilepsy, pneumonia, and respiratory infections, while also identifying strategies to ensure equitable care for people from minoritized ethnic groups and those living in deprived areas.

## Notes

Supplement 1. eTable 1. List of avoidable deaths as defined by the Organisation for Economic Co-operation and Development /Eurostat eTable 2. Co-occurring health conditions by presence of Down syndrome eTable 3. Most common causes of avoidable death, stratified by Down syndrome status and age groups eTable 4. Full multiple logistic regression results for factors associated with avoidable death in adults with Down syndrome eTable 5. Full log-binomial model results for factors associated with avoidable death in adults with Down syndrome eTable 6. Full Cox regression model results for factors associated with age at death among adults with Down syndrome with avoidable deaths eTable 7. Full multiple logistic regression results for factors associated with avoidable death in adults with Down syndrome (for those died before COVID-19 only) eTable 8. Full multiple logistic regression results for factors associated with avoidable death in adults with Down syndrome (for those died during COVID-19 only) eTable 9. Full Cox regression model results for factors associated with age at death among adults with Down syndrome with avoidable deaths (for those died before COVID-19 only) eTable 10. Full Cox regression model results for factors associated with age at death among adults with Down syndrome with avoidable deaths (for those died during COVID-19 only) eFigure 1. Flow diagram eFigure 2. Survival curve by intellectual disability level and Down syndrome status

Supplement 2. Data Sharing Statement
