# Supplementary material for: Avoidable Mortality in Adults With Down Syndrome or With Other Intellectual Disabilities
Source: JAMA Netw Open. 2026 Nov 11;9(11):e2643229. doi: 10.1001/jamanetworkopen.2026.43229 (PMC13756131; doi:10.1001/jamanetworkopen.2026.43229)
Supplement: Supplementary file 1 [file jamanetwopen-e2643229-s001.pdf]

Supplemental Online Content

Bokor L, Yu ML, Carter B, Chauhan U, Sheehan R, Strydom A. Avoidable mortality in adults with down syndrome or with other intellectual disabilities. *JAMA Netw Open.* 2026;9(11):e2643229. doi:10.1001/jamanetworkopen.2026.43229

eTable 1. List of avoidable deaths as defined by the Organisation for Economic Co-operation and Development /Eurostat

eTable 2. Co-occurring health conditions by presence of Down syndrome

eTable 3. Most common causes of avoidable death, stratified by Down syndrome status and age groups

eTable 4. Full multiple logistic regression results for factors associated with avoidable death in adults with Down syndrome

eTable 5. Full log-binomial model results for factors associated with avoidable death in adults with Down syndrome

eTable 6. Full Cox regression model results for factors associated with age at death among adults with Down syndrome with avoidable deaths

eTable 7. Full multiple logistic regression results for factors associated with avoidable death in adults with Down syndrome (for those died before COVID-19 only)

eTable 8. Full multiple logistic regression results for factors associated with avoidable death in adults with Down syndrome (for those died during COVID-19 only)

eTable 9. Full Cox regression model results for factors associated with age at death among adults with Down syndrome with avoidable deaths (for those died before COVID-19 only)

eTable 10. Full Cox regression model results for factors associated with age at death among adults with Down syndrome with avoidable deaths (for those died during COVID-19 only)

eFigure 1. Flow diagram

eFigure 2. Survival curve by intellectual disability level and Down syndrome status

This supplemental material has been provided by the authors to give readers additional information about their work.

© 2026 Bokor L et al. *JAMA Network Open*.

eTable 1. List of avoidable deaths as defined by the Organisation for Economic Co-operation and Development /Eurostat

Condition group and cause	ICD–10 codes	Age	Treatable	Preventable
**Infectious diseases**				
Intestinal diseases	A00–A09	0–74		•
Diphtheria, Tetanus, Poliomyelitis	A35, A36, A80	0–74		•
Whooping cough	A37	0–74		•
Meningococcal infection	A39	0–74		•
Sepsis due to streptococcus pneumonia and sepsis due to haemophilus influenzae	A40.3, A41.3	0–74		•
Haemophilus influenza infections	A49.2	0–74		•
Sexually transmitted infections (except HIV/AIDS)	A50–A60, A63, A64	0–74		•
Varicella	B01	0–74		•
Measles	B05	0–74		•
Rubella	B06	0–74		•
Viral Hepatitis	B15–B19	0–74		•
HIV/AIDS	B20–B24	0–74		•
Malaria	B50–B54	0–74		•
Haemophilus and pneumococcal meningitis	G00.0, G00.1	0–74		•
Tuberculosis	A15–A19, B90, J65	0–74	• (50%)	• (50%)
Scarlet fever	A38	0–74	•	
Sepsis	A40 (excl. A40.3), A41 (excl. A41.3)	0–74	•	
Cellulitis	A46, L03	0–74	•	
Legionnaires disease	A48.1	0–74	•	
Streptococcal and enterococci infection	A49.1	0–74	•	
Other meningitis	G00.2, G00.3, G00.8, G00.9	0–74	•	
Meningitis due to other and unspecified causes	G03	0–74	•	
**Neoplasms**				
Lip, oral cavity and pharynx cancer	C00–C14	0–74		•
Oesophageal cancer	C15	0–74		•
Stomach cancer	C16	0–74		•
Liver cancer	C22	0–74		•
Lung cancer	C33–C34	0–74		•
Mesothelioma	C45	0–74		•
Skin (melanoma) cancer	C43	0–74		•
Bladder cancer	C67	0–74		•
Cervical cancer	C53	0–74	• (50%)	• (50%)
Colorectal cancer	C18–C21	0–74	•	
Breast cancer (female only)	C50	0–74	•	
Uterus cancer	C54, C55	0–74	•	
Testicular cancer	C62	0–74	•	
Thyroid cancer	C73	0–74	•	
Hodgkin's disease	C81	0–74	•	
Lymphoid leukaemia	C91.0, C91.1	0–74	•	
Benign neoplasm	D10–D36	0–74	•	
**Endocrine and metabolic diseases**				
Nutritional deficiency anaemia	D50–D53	0–74		•
Diabetes mellitus	E10–E14	0–74	• (50%)	• (50%)
Thyroid diseases	E00–E07	0–74	•	
Adrenal diseases	E24–E25 (excl E24.4), E27	0–74	•	
**Diseases of the nervous system**				
Epilepsy	G40, G41	0–74	•	
**Diseases of the circulatory system**				

© 2026 Bokor L et al. *JAMA Network Open*.

Condition group and cause	ICD–10 codes	Age	Treatable	Preventable
Aortic aneurysm	I71	0–74	• (50%)	• (50%)
Hypertensive diseases	I10–I13, I15	0–74	• (50%)	• (50%)
Ischaemic heart diseases	I20–I25	0–74	• (50%)	• (50%)
Cerebrovascular diseases	I60–I69	0–74	• (50%)	• (50%)
Other atherosclerosis	I70, I73.9	0–74	• (50%)	• (50%)
Rheumatic and other heart diseases	I00–I09	0–74	•	
Venous thromboembolism	I26, I80, I82.9	0–74	•	
**Diseases of the respiratory system**				
Influenza	J09–J11	0–74		•
Pneumonia due to streptococcus pneumonia or haemophilus influenza	J13–J14	0–74		•
Chronic lower respiratory diseases	J40–J44	0–74		•
Lung diseases due to external agents	J60–J64, J66–J70, J82, J92	0–74		•
Upper respiratory infections	J00–J06, J30–J39	0–74	•	
Pneumonia, not elsewhere classified or organism unspecified	J12, J15, J16–J18	0–74	•	
Acute lower respiratory infections	J20–J22	0–74	•	
Asthma and bronchiectasis	J45–J47	0–74	•	
Adult respiratory distress syndrome	J80	0–74	•	
Pulmonary oedema	J81	0–74	•	
Abscess of lung and mediastinum pyothorax	J85, J86	0–74	•	
Other pleural diseases	J90, J93, J94	0–74	•	
**Diseases of the digestive system**				
Gastric and duodenal ulcer	K25–K28	0–74	•	
Appendicitis	K35–K38	0–74	•	
Abdominal hernia	K40–K46	0–74	•	
Cholelithiasis and cholecystitis	K80–K81	0–74	•	
Other diseases of gallbladder or biliary tract	K82–K83	0–74	•	
Acute pancreatitis	K85.0, K85.1, K85.3, K85.8, K85.9	0–74	•	
Other diseases of pancreas	K86.1, K86.2, K86.3, K86.8, K86.9	0–74	•	
**Diseases of the genitourinary system**				
Nephritis and nephrosis	N00–N07	0–74	•	
Obstructive uropathy	N13, N20–N21, N35	0–74	•	
Renal failure	N17–N19	0–74	•	
Renal colic	N23	0–74	•	
Diseases resulting from renal tubular dysfunction	N25	0–74	•	
Unspecified contracted kidney, small kidney of unknown cause	N26–N27	0–74	•	
Inflammatory diseases of genitourinary system	N34.1, N70–N73, N75.0, N75.1, N76.4, N76.6	0–74	•	
Prostatic hyperplasia	N40	0–74	•	
**Pregnancy, childbirth and the perinatal period**				
Tetanus neonatorum	A33	0–74		•
Obstetrical tetanus	A34	0–74		•
Pregnancy, childbirth and the puerperium	O00–O99	0–74	•	
Certain conditions originating in the perinatal period	P00–P96	0–74	•	
**Congenital malformations**				
Certain congenital malformations (neural tube defects)	Q00, Q01, Q05	0–74		•
Congenital malformations of the circulatory system (heart defects)	Q20–Q28	0–74	•	
**Adverse effects of medical and surgical care**				

© 2026 Bokor L et al. *JAMA Network Open*.

Condition group and cause	ICD–10 codes	Age	Treatable	Preventable
Drugs, medicaments and biological substances causing adverse effects in therapeutic use	Y40–Y59	0–74	•	
Misadventures to patients during surgical and medical care	Y60–Y69, Y83–Y84	0–74	•	
Medical devices associated with adverse incidents in diagnostic and therapeutic use	Y70–Y82	0–74	•	
**Injuries**				
Transport Accidents	V01–V99	0–74		•
Accidental Injuries	W00–X39, X46–X59	0–74		•
Intentional self–harm	X66–X84	0–74		•
Event of undetermined intent	Y16–Y34	0–74		•
Assault	X86–Y09	0–74		•
**Alcohol–related and drug–related deaths**				
Alcohol–specific diseases and poisonings	E24.4, F10, G31.2, G62.1, G72.1, I42.6, K29.2, K70, K85.2, K86.0, Q86.0, R78.0, X45, X65, Y15	0–74		•
Other alcohol–related diseases	K73, K74.0–K74.2, K74.6	0–74		•
Drug diseases and poisonings	F11–F16, F18–F19, X40–X44, X85, Y10–Y14	0–74		•
Intentional self–poisoning by drugs	X60–X64	0–74		•
**Provisional assignment of new diseases**				
COVID–19	U07.1–U07.2	0–74		•

Source: Organisation for Economic Co-operation and Development (1)

© 2026 Bokor L et al. *JAMA Network Open*.

eTable 2. Co-occurring health conditions by presence of Down syndrome

Long-term conditions	Adults with ID with DS (N=1,177) (%)	Adults with ID without DS (N=5,485) (%)
**Physical conditions**		
Dementia	742 (63.0)	534 (9.7)
Dysphagia	712 (60.5)	2,618 (47.7)
Visual problems	428 (36.4)	1,611 (29.4)
Hearing problems	263 (22.3)	795 (14.5)
Kidney problems	233 (19.8)	1,259 (23.0)
Epilepsy	168 (14.3)	629 (11.5)
Diabetes	142 (12.1)	1,201 (21.9)
Hypertension	102 (8.7)	1,235 (22.5)
Asthma	91 (7.7)	668 (12.2)
Deep vein thrombosis	50 (4.2)	211 (3.8)
Osteoporosis	43 (3.7)	293 (5.3)
Cancer	29 (2.5)	382 (7.0)
Stroke	25 (2.1)	178 (3.2)
Chronic obstructive pulmonary disease	16 (1.4)	353 (6.4)
Coronary artery disease	7 (0.6)	56 (1.0)
Peripheral artery disease	5 (0.4)	14 (0.3)
**Mental conditions**		
Anxiety	256 (21.8)	1,468 (26.8)
Depression	188 (16.0)	990 (18.0)
Psychosis	46 (3.9)	564 (10.3)
Bipolar	12 (1.0)	247 (4.5)

Abbreviations: DS, Down syndrome; ID, intellectual disability.

© 2026 Bokor L et al. *JAMA Network Open*.

eTable 3. Most common causes of avoidable death, stratified by Down syndrome status and age groups

Age group and cause of death	Group 1: Adults with ID with DS (N=3,381) (%)	Group 2: Adults with ID without DS (N=15,347) (%)	Group 3: General adult population (N=4,163,160) (%)	*P* Value (1 vs 2)	*P* Value (1 vs 3)
**Aged 18–24**					
All avoidable deaths	15 (100.0)	228 (100.0)	7,183 (100.0)		
COVID–19	NA	28 (12.3)	107 (1.5)	.39	< .001
Congenital malformations of the circulatory system (heart defects)	NA	9 (3.9)	88 (1.2)	.005	< .001
Epilepsy	NA	56 (24.6)	345 (4.8)	.69	.006
Other pleural diseases	NA	0 (0.0)	NA	< .001	< .001
Sepsis	NA	NA	26 (0.4)	< .001	< .001
Asthma and bronchiectasis	NA	8 (3.5)	67 (0.9)	.003	< .001
**Aged 25–49**					
All avoidable deaths	247 (100.0)	1,399 (100.0)	127,111 (100.0)		
COVID–19	71 (28.7)	183 (13.1)	3,998 (3.1)	< .001	< .001
Epilepsy	35 (14.2)	191 (13.7)	1,883 (1.5)	.83	< .001
Congenital malformations of the circulatory system (heart defects)	30 (12.1)	32 (2.3)	680 (0.5)	< .001	< .001
Pneumonia, not elsewhere classified or organism unspecified	20 (8.1)	183 (13.1)	2,821 (2.2)	.03	< .001
Cerebrovascular diseases	15 (6.1)	53 (3.8)	4,712 (3.7)	.10	.05
Asthma and bronchiectasis	6 (2.4)	34 (2.4)	668 (0.5)	.999	< .001
**Aged 50–64**					
All avoidable deaths	1,052 (100.0)	2,751 (100.0)	318,913 (100.0)		
Epilepsy	214 (20.3)	149 (5.4)	1,336 (0.4)	< .001	< .001
COVID–19	209 (19.9)	352 (12.8)	15,483 (4.9)	< .001	< .001
Pneumonia, not elsewhere classified or organism unspecified	137 (13.0)	365 (13.3)	8,371 (2.6)	.84	< .001
Cerebrovascular diseases	85 (8.1)	187 (6.8)	15,544 (4.9)	.17	< .001
Lung diseases due to external agents	52 (4.9)	91 (3.3)	845 (0.3)	.02	< .001
Ischaemic heart diseases	43 (4.1)	296 (10.8)	57,323 (18.0)	< .001	< .001
**Aged 65+**					
All avoidable deaths	275 (100.0)	2,507 (100.0)	461,659 (100.0)		
COVID–19	53 (19.3)	407 (16.2)	25,216 (5.5)	.20	< .001
Pneumonia, not elsewhere classified or organism unspecified	45 (16.4)	395 (15.8)	15,574 (3.4)	.79	< .001
Epilepsy	39 (14.2)	74 (3.0)	793 (0.2)	< .001	< .001
Cerebrovascular diseases	20 (7.3)	218 (8.7)	28,561 (6.1)	.42	.46
Ischaemic heart diseases	17 (6.2)	279 (11.1)	83,737 (18.1)	.012	< .001
Acute lower respiratory infections	13 (4.7)	62 (2.5)	1,961 (0.4)	.03	< .001
**All adults (18+)**					
All avoidable deaths	1,589 (100.0)	6,885 (100.0)	914,866 (100.0)		
COVID–19	336 (21.1)	970 (14.1)	44,804 (4.9)	< .001	< .001
Epilepsy	290 (18.3)	470 (6.8)	4,357 (0.5)	< .001	< .001
Pneumonia, not elsewhere classified or organism unspecified	203 (12.8)	980 (14.2)	26,883 (2.9)	.13	< .001
Cerebrovascular diseases	121 (7.6)	470 (6.8)	48,926 (5.3)	.27	< .001
Lung diseases due to external agents	65 (4.1)	271 (3.9)	2,898 (0.3)	.78	< .001
Ischaemic heart diseases	64 (4.0)	650 (9.4)	152,335 (16.7)	< .001	< .001

Data are presented as numbers (percentages of avoidable deaths in each age group).
Abbreviations: DS, Down syndrome; ID, intellectual disability.
NA: Suppressed for those <5 cases. <5 cases are imputed as 3 cases for statistical test.

© 2026 Bokor L et al. *JAMA Network Open*.

eTable 4. Full multiple logistic regression results for factors associated with avoidable death in adults with Down syndrome

Predictors	Unadjusted odds ratio (95% CI)	*P* Value	Adjusted odds ratio (95% CI)[a]	*P* Value
**All adults with intellectual disabilities (n=18,728)**				
Down Syndrome Status				
Without Down syndrome (reference)	NA	NA	NA	NA
With Down syndrome	1.09 (1.01–1.17)	.02	1.15 (1.06–1.25)	< .001
Ethnicity				
White (reference)	NA	NA	NA	NA
Ethnic groups other than White	1.26 (1.12–1.42)	< .001	1.23 (1.09–1.40)	< .001
Index of Multiple Deprivation (IMD)				
9–10 (least deprived, reference)	NA	NA	NA	NA
7–8	1.10 (0.98–1.23)	.10	1.04 (0.92–1.17)	.57
5–6	1.15 (1.04–1.28)	.009	1.12 (1.00–1.25)	.06
3–4	1.14 (1.03–1.27)	.012	1.06 (0.94–1.18)	.35
1–2 (most deprived)	1.25 (1.13–1.39)	< .001	1.20 (1.07–1.33)	.001
Sex				
Female (reference)	NA	NA	NA	NA
Male	1.05 (0.99–1.11)	.12	1.04 (0.97–1.10)	.27
COVID–19 period[b]				
Before COVID–19 (reference)	NA	NA	NA	NA
During COVID–19	1.03 (0.97–1.09)	.39	1.03 (0.96–1.11)	.36
After COVID–19	0.44 (0.39–0.50)	< .001	0.44 (0.38–0.50)	< .001
**Adults with Down syndrome (n=3,381)**				
Ethnicity				
White (reference)	NA	NA	NA	NA
Ethnic groups other than White	1.61 (1.16–2.24)	.005	1.56 (1.11–2.18)	.01
Index of Multiple Deprivation (IMD)				
9–10 (least deprived, reference)	NA	NA	NA	NA
7–8	0.96 (0.75–1.23)	.76	0.86 (0.66–1.12)	.26
5–6	1.12 (0.88–1.42)	.37	1.03 (0.80–1.34)	.81
3–4	1.09 (0.87–1.38)	.45	1.02 (0.79–1.31)	.90
1–2 (most deprived)	1.11 (0.88–1.41)	.39	1.03 (0.80–1.34)	.81
Sex				
Female (reference)	NA	NA	NA	NA
Male	0.99 (0.86–1.13)	.87	0.98 (0.85–1.14)	.82
COVID–19 period[b]				
Before COVID–19 (reference)	NA	NA	NA	NA
During COVID–19	1.42 (1.23–1.64)	< .001	1.39 (1.18–1.63)	< .001
After COVID–19	0.45 (0.33–0.61)	< .001	0.42 (0.30–0.59)	< .001
**Adults with Down syndrome with coded data (n=1,177)**				
Ethnicity				
White (reference)	NA	NA	NA	NA
Ethnic groups other than White	1.47 (0.86–2.53)	.16	1.41 (0.81–2.45)	.23
Index of Multiple Deprivation (IMD)				
9–10 (least deprived, reference)	NA	NA	NA	NA
7–8	0.95 (0.62–1.45)	.81	0.97 (0.62–1.50)	.88
5–6	1.08 (0.71–1.62)	.73	1.21 (0.79–1.85)	.39
3–4	1.04 (0.70–1.54)	.86	1.09 (0.72–1.65)	.67
1–2 (most deprived)	1.17 (0.78–1.75)	.45	1.29 (0.85–1.95)	.23
Sex				
Female (reference)	NA	NA	NA	NA
Male	0.85 (0.67–1.07)	.16	0.84 (0.66–1.07)	.15
COVID–19 period[b]				

© 2026 Bokor L et al. *JAMA Network Open*.

Predictors	Unadjusted odds ratio (95% CI)	*P* Value	Adjusted odds ratio (95% CI)[a]	*P* Value
Before COVID–19	NA	NA	NA	NA
During COVID–19 (reference)	NA	NA	NA	NA
After COVID–19	0.32 (0.22–0.45)	< .001	0.30 (0.21–0.43)	< .001
Physical health conditions				
Osteoporosis	2.53 (1.32–4.83)	.005	2.12 (1.08–4.15)	.03
Diabetes	1.67 (1.17–2.38)	.005	1.72 (1.19–2.49)	.004
Asthma	1.80 (1.17–2.78)	.008	1.64 (1.05–2.57)	.03
Dementia	0.54 (0.43–0.69)	< .001	0.54 (0.42–0.69)	< .001
Epilepsy	0.46 (0.33–0.66)	< .001	0.74 (0.50–1.10)	.14
Stroke	1.09 (0.49–2.41)	.83	1.85 (0.78–4.39)	.17
Peripheral artery disease	0.79 (0.13–4.72)	.79	1.49 (0.23–9.72)	.68
Deep vein thrombosis	1.09 (0.62–1.93)	.76	1.10 (0.61–1.96)	.76
Hearing problems	1.01 (0.76–1.33)	.96	1.05 (0.79–1.40)	.73
Kidney problems	1.02 (0.77–1.37)	.87	1.02 (0.75–1.38)	.90
Chronic obstructive pulmonary disease	0.92 (0.34–2.48)	.86	0.97 (0.34–2.71)	.95
Hypertension	0.85 (0.56–1.28)	.43	0.90 (0.58–1.38)	.63
Visual problems	0.90 (0.71–1.14)	.37	0.89 (0.69–1.14)	.35
Cancer	0.61 (0.28–1.33)	.22	0.86 (0.38–1.95)	.71
Dysphagia	0.94 (0.74–1.18)	.58	0.81 (0.63–1.04)	.09
Coronary artery disease	NA	NA	NA	NA
Peripheral artery disease	NA	NA	NA	NA
Mental health conditions				
Bipolar	0.84 (0.27–2.66)	.77	1.19 (0.33–4.33)	.79
Depression	0.85 (0.62–1.17)	.32	1.09 (0.79–1.52)	.60
Anxiety	0.91 (0.69–1.21)	.53	0.92 (0.69–1.23)	.56
Psychosis	0.62 (0.33–1.15)	.13	0.61 (0.32–1.15)	.13

Abbreviations: CI, confidence interval

[a]Adjusted by sex, ethnicity, index of multiple deprivation, COVID–19 period, and Down syndrome where appropriate.

[b]Defined by the World Health Organization as the period of the COVID–19 Public Health Emergency of International Concern: 30 January 2020 to 5 May 2023. (2)

NA: Suppressed for those <5 cases

© 2026 Bokor L et al. *JAMA Network Open*.

eTable 5. Full log-binomial model results for factors associated with avoidable death in adults with Down syndrome

Predictors	Unadjusted risk ratios (95% CI)	*P* Value	Adjusted risk ratios (95% CI)[b]	*P* Value
**All adults with intellectual disabilities (n=18,728)**				
Down Syndrome Status				
Without Down syndrome (reference)	NA	NA	NA	NA
With Down syndrome	1.07 (1.03-1.12)	.001	1.08 (1.04-1.13)	<.001
Ethnicity				
White (reference)	NA	NA	NA	NA
Ethnic groups other than White	1.13 (1.06-1.20)	<.001	1.11 (1.05-1.18)	<.001
Index of Multiple Deprivation (IMD)				
9–10 (least deprived, reference)	NA	NA	NA	NA
7–8	1.03 (0.96-1.10)	.43	1.02 (0.95-1.09)	.58
5–6	1.06 (1.00-1.13)	.06	1.06 (1.00-1.13)	.07
3–4	1.04 (0.97-1.11)	.25	1.03 (0.97-1.10)	.36
1–2 (most deprived)	1.11 (1.04-1.18)	.001	1.10 (1.04-1.17)	.002
Sex				
Female (reference)	NA	NA	NA	NA
Male	1.02 (0.98-1.05)	.30	1.02 (0.99-1.06)	.24
COVID–19 period[c]				
Before COVID–19 (reference)	NA	NA	NA	NA
During COVID–19	1.02 (0.98-1.06)	.33	1.02 (0.98-1.06)	.28
After COVID–19	0.59 (0.54-0.65)	<.001	0.59 (0.54-0.65)	<.001
**Adults with Down syndrome (n=3,381)**				
Ethnicity				
White (reference)	NA	NA	NA	NA
Ethnic groups other than White	1.26 (1.10-1.45)	.001	1.21 (1.06-1.39)	.006
Index of Multiple Deprivation (IMD)				
9–10 (least deprived, reference)	NA	NA	NA	NA
7–8	0.93 (0.80-1.07)	.29	0.92 (0.80-1.05)	.22
5–6	1.01 (0.88-1.15)	.92	1.02 (0.90-1.16)	.75
3–4	1.00 (0.88-1.14)	>.99	1.00 (0.88-1.13)	.96
1–2 (most deprived)	1.02 (0.89-1.16)	.78	1.01 (0.89-1.15)	.86
Sex				
Female (reference)	NA	NA	NA	NA
Male	0.99 (0.91-1.07)	.76	0.99 (0.92-1.07)	.82
COVID–19 period [c]				
Before COVID–19 (reference)	NA	NA	NA	NA
During COVID–19	1.19 (1.09-1.30)	<.001	1.18 (1.08-1.29)	<.001
After COVID–19	0.57 (0.45-0.73)	<.001	0.57 (0.45-0.72)	<.001
**Adults with Down syndrome with coded data (n=1,177)[a]**				
Ethnicity				
White (reference)	NA	NA	NA	NA
Ethnic groups other than White	1.21 (0.95-1.54)	.13	1.10 (0.85-1.41)	.46
Index of Multiple Deprivation (IMD)				
9–10 (least deprived, reference)	NA	NA	NA	NA
7–8	1.01 (0.79-1.28)	.95	1.00 (0.79-1.27)	.99
5–6	1.08 (0.85-1.35)	.54	1.08 (0.87-1.35)	.49
3–4	1.04 (0.83-1.30)	.74	1.02 (0.82-1.27)	.87
1–2 (most deprived)	1.14 (0.91-1.42)	.25	1.10 (0.88-1.36)	.40
Sex				
Female (reference)	NA	NA	NA	NA
Male	0.91 (0.81-1.04)	.16	0.92 (0.81-1.04)	.17
COVID–19 period[c]				

© 2026 Bokor L et al. *JAMA Network Open*.

Predictors	Unadjusted risk ratios (95% CI)	*P* Value	Adjusted risk ratios (95% CI)[b]	*P* Value
Before COVID–19	NA	NA	NA	NA
During COVID–19 (reference)[d]	NA	NA	NA	NA
After COVID–19	0.47 (0.36-0.61)	<.001	0.51 (0.38-0.67)	<.001
Physical health conditions				
Osteoporosis	1.50 (1.21-1.87)	<.001	1.28 (1.02-1.62)	.03
Diabetes	1.29 (1.11-1.51)	.001	1.24 (1.07-1.45)	.006
Asthma	1.32 (1.10-1.58)	.003	1.17 (0.98-1.41)	.09
Dementia	0.73 (0.65-0.83)	<.001	0.75 (0.66-0.85)	<.001
Epilepsy	0.62 (0.49-0.79)	<.001	0.85 (0.66-1.10)	.21
Stroke	1.08 (0.72-1.62)	.71	1.53 (1.01-2.34)	.046
Deep vein thrombosis	1.04 (0.77-1.39)	.81	1.14 (0.85-1.54)	.39
Hearing problems	1.01 (0.87-1.17)	.89	1.04 (0.90-1.21)	.60
Kidney problems	0.99 (0.85-1.16)	.91	0.95 (0.82-1.12)	.56
Chronic obstructive pulmonary disease	0.94 (0.54-1.65)	.84	0.91 (0.53-1.56)	.74
Hypertension	0.91 (0.72-1.15)	.43	0.87 (0.69-1.09)	.22
Visual problems	0.95 (0.83-1.08)	.41	0.93 (0.82-1.07)	.31
Cancer	0.74 (0.45-1.23)	.24	0.81 (0.49-1.35)	.42
Dysphagia	0.96 (0.85-1.09)	.57	1.00 (0.88-1.15)	.95
Coronary artery disease	NA	NA	NA	NA
Peripheral artery disease	NA	NA	NA	NA
Mental health conditions				
Bipolar	1.08 (0.58-2.01)	.81	1.07 (0.55-2.06)	.84
Depression	0.96 (0.81-1.15)	.69	0.97 (0.81-1.15)	.70
Anxiety	0.96 (0.82-1.12)	.58	0.98 (0.84-1.14)	.76
Psychosis	0.74 (0.50-1.11)	.15	0.76 (0.50-1.14)	.18

Abbreviations: CI, confidence interval; NA, not applicable.

[a] Modified Poisson regression with robust (sandwich) variance estimation was used for this panel because the log-binomial model did not converge.

[b]Adjusted by sex, ethnicity, index of multiple deprivation, COVID–19 period, and Down syndrome where appropriate.

[c]Defined by the World Health Organization as the period of the COVID–19 Public Health Emergency of International Concern: 30 January 2020 to 5 May 2023. (2)

[d]Coded data on co-occurring conditions were available only for reviews completed 2021 onwards. This panel therefore contains no deaths from before COVID-19 period, and the during-pandemic period is the reference category.

NA: Suppressed for those <5 cases

© 2026 Bokor L et al. *JAMA Network Open*.

eTable 6. Full Cox regression model results for factors associated with age at death among adults with Down syndrome with avoidable deaths

Predictors	Unadjusted hazard ratio (95% CI)	*P* Value	Adjusted hazard ratio (95% CI)[a]	*P* Value
**All adults with intellectual disabilities with avoidable deaths (n=8,474)**				
Down syndrome status				
Without Down syndrome (reference)	NA	NA	NA	NA
With Down syndrome	1.46 (1.38–1.54)	< .001	1.49 (1.40–1.58)	< .001
Ethnicity				
White (reference)	NA	NA	NA	NA
Ethnic groups other than White	2.26 (2.07–2.46)	< .001	2.31 (2.11–2.52)	< .001
Index of Multiple Deprivation (IMD)				
9–10 (least deprived, reference)	NA	NA	NA	NA
7–8	0.98 (0.91–1.07)	.70	0.98 (0.90–1.07)	.61
5–6	1.03 (0.95–1.11)	.55	1.03 (0.94–1.12)	.57
3–4	1.03 (0.95–1.12)	.45	1.00 (0.92–1.09)	.998
1–2 (most deprived)	1.04 (0.97–1.13)	.27	1.04 (0.96–1.13)	.36
Sex				
Female (reference)	NA	NA	NA	NA
Male	0.93 (0.89–0.97)	< .001	0.94 (0.90–0.99)	.014
COVID–19 period [b]				
Before COVID–19 (reference)	NA	NA	NA	NA
During COVID–19	0.95 (0.90–0.99)	.02	0.95 (0.90–1.00)	.04
After COVID–19	0.98 (0.88–1.09)	.66	0.97 (0.87–1.09)	.59
**Adults with Down syndrome with avoidable deaths (n=1,589)**				
Ethnicity				
White (reference)	NA	NA	NA	NA
Ethnic groups other than White	2.10 (1.69–2.60)	< .001	2.11 (1.70–2.61)	< .001
Index of Multiple Deprivation (IMD)				
9–10 (least deprived, reference)	NA	NA	NA	NA
7–8	1.08 (0.90–1.30)	.42	1.03 (0.85–1.26)	.74
5–6	1.16 (0.98–1.39)	.09	1.13 (0.94–1.36)	.20
3–4	1.14 (0.96–1.36)	.14	1.12 (0.93–1.34)	.24
1–2 (most deprived)	1.28 (1.07–1.52)	.006	1.25 (1.04–1.51)	.02
Sex				
Female (reference)	NA	NA	NA	NA
Male	1.04 (0.94–1.15)	.46	1.05 (0.94–1.17)	.36
COVID–19 period [b]				
Before COVID–19 (reference)	NA	NA	NA	NA
During COVID–19	1.11 (1.00–1.23)	.06	1.10 (0.98–1.24)	.12
After COVID–19	1.00 (0.77–1.30)	.98	0.93 (0.70–1.23)	.59
**Adults with Down syndrome with avoidable deaths and coded data (n=540)**				
Ethnicity				
White (reference)	NA	NA	NA	NA
Ethnic groups other than White	2.08 (1.45–2.99)	< .001	2.22 (1.53–3.23)	< .001
Index of Multiple Deprivation (IMD)				
9–10 (least deprived, reference)	NA	NA	NA	NA
7–8	1.09 (0.79–1.50)	.61	1.12 (0.81–1.55)	.50
5–6	1.14 (0.84–1.54)	.41	1.10 (0.81–1.51)	.54
3–4	1.05 (0.78–1.42)	.74	1.05 (0.78–1.43)	.73
1–2 (most deprived)	1.37 (1.02–1.85)	.04	1.38 (1.02–1.88)	.04
Sex				
Female (reference)	NA	NA	NA	NA
Male	1.05 (0.89–1.25)	.57	1.03 (0.88–1.22)	.74
COVID–19 period [b]				

© 2026 Bokor L et al. *JAMA Network Open*.

Predictors	Unadjusted hazard ratio (95% CI)	*P* Value	Adjusted hazard ratio (95% CI)[a]	*P* Value
Before COVID–19	NA	NA	NA	NA
During COVID–19 (reference)[c]	NA	NA	NA	NA
After COVID–19	0.91 (0.67–1.23)	.53	0.83 (0.60–1.14)	.24
Physical health conditions				
Dementia	0.74 (0.62–0.87)	< .001	0.75 (0.63–0.90)	.002
Kidney problems	0.81 (0.66–1.01)	.06	0.79 (0.63–0.98)	.04
Dysphagia	0.82 (0.69–0.98)	.03	0.82 (0.69–0.98)	.03
Cancer	1.42 (0.76–2.67)	.27	1.52 (0.80–2.88)	.20
Asthma	1.15 (0.87–1.53)	.33	1.11 (0.83–1.48)	.49
Peripheral artery disease	0.91 (0.23–3.65)	.89	1.08 (0.27–4.42)	.91
Visual problems	1.04 (0.87–1.24)	.69	1.06 (0.89–1.28)	.52
Deep vein thrombosis	0.99 (0.66–1.49)	.96	1.01 (0.66–1.53)	.98
Hearing problems	0.98 (0.80–1.20)	.87	0.99 (0.81–1.22)	.96
Chronic obstructive pulmonary disease	0.90 (0.43–1.91)	.79	0.97 (0.46–2.08)	.94
Hypertension	1.00 (0.73–1.37)	.996	0.92 (0.66–1.27)	.60
Osteoporosis	0.97 (0.67–1.42)	.89	0.89 (0.61–1.31)	.56
Diabetes	0.88 (0.70–1.12)	.30	0.86 (0.68–1.09)	.22
Epilepsy	0.76 (0.57–1.02)	.07	0.77 (0.56–1.07)	.12
Stroke	0.73 (0.41–1.29)	.28	0.68 (0.37–1.24)	.21
Coronary artery disease	NA	NA	NA	NA
Peripheral artery disease	NA	NA	NA	NA
Mental health conditions				
Anxiety	1.15 (0.94–1.42)	.18	1.20 (0.96–1.48)	.11
Depression	1.16 (0.92–1.48)	.22	1.16 (0.91–1.48)	.24
Bipolar	1.21 (0.50–2.91)	.68	1.14 (0.47–2.78)	.77
Psychosis	0.95 (0.58–1.56)	.83	0.91 (0.55–1.51)	.72
**Adults with Down syndrome with avoidable deaths, coded data and dementia (n=299)**				
Kidney problems	0.83 (0.62–1.11)	.22	0.85 (0.63–1.15)	.29
Dysphagia	1.11 (0.86–1.44)	.42	1.13 (0.86–1.46)	.38

Abbreviations: CI, confidence interval; NA, not applicable

[a]Adjusted by sex, ethnicity, index of multiple deprivation, COVID–19 period, and Down syndrome where appropriate.

[b]Defined by the World Health Organization as the period of the COVID–19 Public Health Emergency of International Concern: 30 January 2020 to 5 May 2023. (2)

NA: Suppressed for those <5 cases

eTable 7. Full multiple logistic regression results for factors associated with avoidable death in adults with Down syndrome (for those died before COVID-19 only)

Predictors	Unadjusted odds ratio (95% CI)	*P* Value	Adjusted odds ratio (95% CI)[a]	*P* Value
**All adults with intellectual disabilities (n=6,562)**				
Down Syndrome Status				
Without Down syndrome (reference)	NA	NA	NA	NA
With Down syndrome	0.90 (0.77-1.05)	.17	0.91 (0.78-1.06)	.21
Ethnicity				
White (reference)	NA	NA	NA	NA
Ethnic groups other than White	1.07 (0.83-1.37)	.61	1.05 (0.82-1.35)	.69
Index of Multiple Deprivation (IMD)				
9–10 (least deprived, reference)	NA	NA	NA	NA
7–8	0.98 (0.79-1.22)	.88	0.98 (0.79-1.22)	.89
5–6	0.99 (0.81-1.23)	.96	1.00 (0.81-1.23)	.96
3–4	1.02 (0.83-1.26)	.84	1.02 (0.83-1.25)	.85
1–2 (most deprived)	1.13 (0.92-1.38)	.25	1.12 (0.92-1.37)	.27
Sex				
Female (reference)	NA	NA	NA	NA
Male	0.97 (0.86-1.09)	.57	0.96 (0.86-1.09)	.54
**Adults with Down syndrome (n=1,286)**				
Ethnicity				
White (reference)	NA	NA	NA	NA
Ethnic groups other than White	1.74 (0.81-3.72)	.16	1.88 (0.87-4.05)	.11
Index of Multiple Deprivation (IMD)				
9–10 (least deprived, reference)	NA	NA	NA	NA
7–8	0.73 (0.45-1.18)	.20	0.71 (0.43-1.15)	.16
5–6	0.87 (0.54-1.38)	.54	0.85 (0.54-1.36)	.51
3–4	1.13 (0.71-1.79)	.60	1.11 (0.70-1.76)	.66
1–2 (most deprived)	0.75 (0.47-1.20)	.22	0.72 (0.45-1.16)	.18
Sex				
Female (reference)	NA	NA	NA	NA
Male	0.95 (0.72-1.26)	.74	0.96 (0.73-1.27)	.78

Abbreviations: CI, confidence interval; NA, not applicable.

[a] Adjusted by sex, ethnicity, index of multiple deprivation and Down syndrome where appropriate.

© 2026 Bokor L et al. *JAMA Network Open*.

eTable 8. Full multiple logistic regression results for factors associated with avoidable death in adults with Down syndrome (for those died during COVID-19 only)

Predictors	Unadjusted odds ratio (95% CI)	*P* Value	Adjusted odds ratio (95% CI)[a]	*P* Value
**All adults with intellectual disabilities (n=10,791)**				
Down Syndrome Status				
Without Down syndrome (reference)	NA	NA	NA	NA
With Down syndrome	1.30 (1.18-1.44)	<.001	1.32 (1.19-1.46)	<.001
Ethnicity				
White (reference)	NA	NA	NA	NA
Ethnic groups other than White	1.29 (1.11-1.50)	<.001	1.28 (1.10-1.49)	.001
Index of Multiple Deprivation (IMD)				
9–10 (least deprived, reference)	NA	NA	NA	NA
7–8	1.07 (0.92-1.24)	.38	1.07 (0.93-1.24)	.36
5–6	1.16 (1.01-1.34)	.04	1.17 (1.01-1.35)	.03
3–4	1.08 (0.94-1.24)	.28	1.07 (0.93-1.24)	.31
1–2 (most deprived)	1.24 (1.08-1.42)	.002	1.24 (1.08-1.43)	.002
Sex				
Female (reference)	NA	NA	NA	NA
Male	1.09 (1.00-1.17)	.04	1.09 (1.01-1.18)	.03
**Adults with Down syndrome (n=1,857)**				
Ethnicity				
White (reference)	NA	NA	NA	NA
Ethnic groups other than White	1.49 (1.01-2.19)	.046	1.47 (0.99-2.17)	.05
Index of Multiple Deprivation (IMD)				
9–10 (least deprived, reference)	NA	NA	NA	NA
7–8	0.87 (0.63-1.21)	.42	0.87 (0.62-1.21)	.41
5–6	1.14 (0.82-1.58)	.43	1.13 (0.82-1.57)	.46
3–4	0.92 (0.67-1.26)	.61	0.91 (0.66-1.25)	.55
1–2 (most deprived)	1.13 (0.82-1.56)	.45	1.11 (0.81-1.53)	.52
Sex				
Female (reference)	NA	NA	NA	NA
Male	1.01 (0.83-1.21)	.94	1.01 (0.84-1.22)	.92
**Adults with Down syndrome with coded data (n=986)**				
Ethnicity				
White (reference)	NA	NA	NA	NA
Ethnic groups other than White	1.26 (0.70-2.27)	.43	1.02 (0.55-1.88)	.96
Index of Multiple Deprivation (IMD)				
9–10 (least deprived, reference)	NA	NA	NA	NA
7–8	0.87 (0.55-1.39)	.57	0.91 (0.57-1.47)	.71
5–6	1.22 (0.78-1.93)	.39	1.21 (0.75-1.93)	.44
3–4	0.95 (0.61-1.47)	.82	0.91 (0.58-1.43)	.68
1–2 (most deprived)	1.12 (0.72-1.74)	.61	1.05 (0.66-1.65)	.85
Sex				
Female (reference)	NA	NA	NA	NA
Male	0.85 (0.66-1.09)	.20	0.84 (0.65-1.10)	.21
Physical health conditions				
Osteoporosis	2.16 (1.10-4.22)	.02	1.93 (0.96-3.88)	.06
Diabetes	2.03 (1.36-3.05)	.001	1.87 (1.22-2.86)	.004
Asthma	1.60 (1.00-2.55)	.05	1.33 (0.81-2.18)	.26
Dementia	0.50 (0.38-0.65)	<.001	0.55 (0.40-0.73)	<.001
Epilepsy	0.60 (0.36-0.99)	.047	0.59 (0.34-1.01)	.05
Stroke	1.96 (0.59-6.55)	.28	2.66 (0.72-9.85)	.14
Deep vein thrombosis	0.83 (0.44-1.56)	.57	0.99 (0.52-1.90)	.98
Hearing problems	1.05 (0.77-1.42)	.77	1.08 (0.77-1.50)	.66

© 2026 Bokor L et al. *JAMA Network Open*.

Predictors	Unadjusted odds ratio (95% CI)	*P* Value	Adjusted odds ratio (95% CI)[a]	*P* Value
Kidney problems	1.09 (0.79-1.51)	.60	0.97 (0.69-1.37)	.86
Chronic obstructive pulmonary disease	0.97 (0.31-3.03)	.96	0.73 (0.22-2.41)	.61
Hypertension	1.09 (0.68-1.74)	.73	0.88 (0.54-1.46)	.63
Visual problems	0.93 (0.71-1.21)	.58	0.92 (0.69-1.22)	.56
Cancer	1.30 (0.45-3.77)	.63	1.06 (0.34-3.34)	.92
Dysphagia	0.73 (0.56-0.95)	.02	0.90 (0.67-1.21)	.49
Coronary artery disease	NA	NA	NA	NA
Peripheral artery disease	NA	NA	NA	NA
Mental health conditions				
Bipolar	0.97 (0.24-3.90)	.97	0.85 (0.20-3.61)	.83
Depression	1.06 (0.75-1.51)	.73	1.08 (0.74-1.59)	.68
Anxiety	0.95 (0.70-1.29)	.75	0.98 (0.71-1.37)	.93
Psychosis	0.61 (0.31-1.20)	.15	0.56 (0.27-1.17)	.12

Abbreviations: CI, confidence interval; NA, not applicable.
[a]Adjusted by sex, ethnicity, index of multiple deprivation and Down syndrome where appropriate.
[b]COVID-19 period is defined by the World Health Organization as the period of the COVID–19 Public Health Emergency of International Concern: 30 January 2020 to 5 May 2023. (2)
Numbers reflect complete cases after exclusion of records with missing sex, ethnicity, or Index of Multiple Deprivation.
NA: Suppressed for those <5 cases

© 2026 Bokor L et al. *JAMA Network Open*.

eTable 9. Full Cox regression model results for factors associated with age at death among adults with Down syndrome with avoidable deaths (for those died before COVID-19 only)

Predictors	Unadjusted hazard ratio (95% CI)	*P* Value	Adjusted hazard ratio (95% CI)[a]	*P* Value
**All adults with intellectual disabilities with avoidable deaths (n=3,035)**				
Down syndrome status				
Without Down syndrome (reference)	NA	NA	NA	NA
With Down syndrome	1.33 (1.18-1.49)	<.001	1.33 (1.19-1.49)	<.001
Ethnicity				
White (reference)	NA	NA	NA	NA
Ethnic groups other than White	2.49 (2.08-2.99)	<.001	2.52 (2.10-3.02)	<.001
Index of Multiple Deprivation (IMD)				
9–10 (least deprived, reference)	NA	NA	NA	NA
7–8	0.94 (0.80-1.11)	.47	0.93 (0.79-1.10)	.41
5–6	0.94 (0.81-1.10)	.45	0.93 (0.80-1.09)	.39
3–4	0.98 (0.84-1.14)	.77	0.96 (0.83-1.12)	.63
1–2 (most deprived)	0.91 (0.79-1.06)	.24	0.91 (0.79-1.06)	.23
Sex				
Female (reference)	NA	NA	NA	NA
Male	0.93 (0.85-1.02)	.12	0.95 (0.87-1.03)	.22
**Adults with Down syndrome with avoidable deaths (n=559)**				
Ethnicity				
White (reference)	NA	NA	NA	NA
Ethnic groups other than White	1.92 (1.16-3.18)	.01	1.98 (1.19-3.29)	.009
Index of Multiple Deprivation (IMD)				
9–10 (least deprived, reference)	NA	NA	NA	NA
7–8	0.90 (0.62-1.29)	.55	0.84 (0.58-1.22)	.37
5–6	1.01 (0.71-1.42)	.97	0.97 (0.69-1.37)	.87
3–4	1.01 (0.72-1.40)	.97	0.98 (0.70-1.36)	.88
1–2 (most deprived)	0.84 (0.59-1.20)	.35	0.83 (0.58-1.18)	.30
Sex				
Female (reference)	NA	NA	NA	NA
Male	1.09 (0.88-1.34)	.43	1.11 (0.90-1.37)	.33

Abbreviations: CI, confidence interval; NA, not applicable.

[a] Adjusted by sex, ethnicity, index of multiple deprivation, and Down syndrome where appropriate.

[b] COVID-19 period is defined by the World Health Organization as the period of the COVID–19 Public Health Emergency of International Concern: 30 January 2020 to 5 May 2023. (2)

© 2026 Bokor L et al. *JAMA Network Open*.

eTable 10. Full Cox regression model results for factors associated with age at death among adults with Down syndrome with avoidable deaths (for those died during COVID-19 only)

Predictors	Unadjusted hazard ratio (95% CI)	*P* Value	Adjusted hazard ratio (95% CI)[a]	*P* Value
**All adults with intellectual disabilities with avoidable deaths (n=5,063)**				
Down syndrome status				
Without Down syndrome (reference)	NA	NA	NA	NA
With Down syndrome	1.55 (1.44-1.67)	<.001	1.58 (1.46-1.70)	<.001
Ethnicity				
White (reference)	NA	NA	NA	NA
Ethnic groups other than White	2.31 (2.09-2.57)	<.001	2.33 (2.10-2.58)	<.001
Index of Multiple Deprivation (IMD)				
9–10 (least deprived, reference)	NA	NA	NA	NA
7–8	0.99 (0.89-1.10)	.84	1.01 (0.90-1.12)	.88
5–6	1.06 (0.95-1.18)	.30	1.08 (0.97-1.20)	.17
3–4	1.06 (0.95-1.17)	.31	1.05 (0.95-1.17)	.32
1–2 (most deprived)	1.08 (0.97-1.19)	.15	1.10 (1.00-1.22)	.06
Sex				
Female (reference)	NA	NA	NA	NA
Male	0.92 (0.87-0.98)	.005	0.94 (0.89-1.00)	.046
**Adults with Down syndrome with avoidable deaths (n=969)**				
Ethnicity				
White (reference)	NA	NA	NA	NA
Ethnic groups other than White	2.30 (1.80-2.94)	<.001	2.30 (1.80-2.94)	<.001
Index of Multiple Deprivation (IMD)				
9–10 (least deprived, reference)	NA	NA	NA	NA
7–8	1.13 (0.89-1.42)	.32	1.13 (0.89-1.42)	.32
5–6	1.23 (0.98-1.54)	.07	1.20 (0.96-1.50)	.12
3–4	1.21 (0.97-1.51)	.09	1.20 (0.96-1.50)	.10
1–2 (most deprived)	1.45 (1.16-1.81)	.001	1.44 (1.16-1.80)	.001
Sex				
Female (reference)	NA	NA	NA	NA
Male	1.04 (0.91-1.18)	.59	1.01 (0.89-1.15)	.84
**Adults with Down syndrome with avoidable deaths and coded data (n=494)**				
Ethnicity				
White (reference)	NA	NA	NA	NA
Ethnic groups other than White	2.57 (1.74-3.81)	<.001	2.58 (1.72-3.88)	<.001
Index of Multiple Deprivation (IMD)				
9–10 (least deprived, reference)	NA	NA	NA	NA
7–8	1.13 (0.81-1.57)	.49	1.10 (0.78-1.56)	.58
5–6	1.15 (0.84-1.59)	.38	1.03 (0.74-1.45)	.84
3–4	1.14 (0.83-1.55)	.42	1.05 (0.75-1.45)	.79
1–2 (most deprived)	1.37 (1.01-1.87)	.046	1.34 (0.97-1.86)	.08
Sex				
Female (reference)	NA	NA	NA	NA
Male	1.03 (0.86-1.23)	.73	1.05 (0.87-1.27)	.64
Physical health conditions				
Osteoporosis	0.96 (0.66-1.41)	.85	0.80 (0.54-1.19)	.27
Diabetes	0.89 (0.70-1.14)	.35	0.86 (0.67-1.12)	.27
Asthma	1.08 (0.80-1.45)	.61	1.11 (0.81-1.52)	.53
Dementia	0.73 (0.61-0.87)	.001	0.73 (0.60-0.88)	.001
Epilepsy	0.74 (0.50-1.10)	.13	0.61 (0.39-0.97)	.04
Stroke	0.95 (0.47-1.90)	.88	1.17 (0.54-2.54)	.68
Deep vein thrombosis	0.98 (0.62-1.55)	.94	0.98 (0.61-1.58)	.94
Hearing problems	1.01 (0.82-1.25)	.92	0.93 (0.74-1.17)	.54

© 2026 Bokor L et al. *JAMA Network Open*.

Predictors	Unadjusted hazard ratio (95% CI)	*P* Value	Adjusted hazard ratio (95% CI)[a]	*P* Value
Kidney problems	0.78 (0.62-0.98)	.04	0.74 (0.58-0.95)	.02
Chronic obstructive pulmonary disease	1.31 (0.58-2.93)	.52	1.08 (0.46-2.50)	.86
Hypertension	0.94 (0.68-1.30)	.73	0.80 (0.56-1.15)	.23
Visual problems	1.04 (0.87-1.26)	.65	1.06 (0.85-1.31)	.61
Cancer	1.17 (0.58-2.35)	.66	1.53 (0.71-3.27)	.27
Dysphagia	0.86 (0.71-1.03)	.09	0.88 (0.72-1.08)	.22
Coronary artery disease	NA	NA	NA	NA
Peripheral artery disease	NA	NA	NA	NA
Mental health conditions				
Anxiety	1.09 (0.41-2.91)	.87	0.92 (0.33-2.61)	.88
Depression	1.15 (0.90-1.46)	.28	1.20 (0.90-1.59)	.21
Bipolar	1.18 (0.95-1.47)	.14	1.26 (1.00-1.60)	.05
Psychosis	0.89 (0.52-1.52)	.68	0.74 (0.42-1.31)	.30

Abbreviations: CI, confidence interval; NA, not applicable.

[a] Adjusted by sex, ethnicity, index of multiple deprivation, and Down syndrome where appropriate.

[b] COVID-19 period is defined by the World Health Organization as the period of the COVID–19 Public Health Emergency of International Concern: 30 January 2020 to 5 May 2023. (2)

NA: Suppressed for those <5 cases

© 2026 Bokor L et al. *JAMA Network Open*.

eFigure 1. Flow diagram

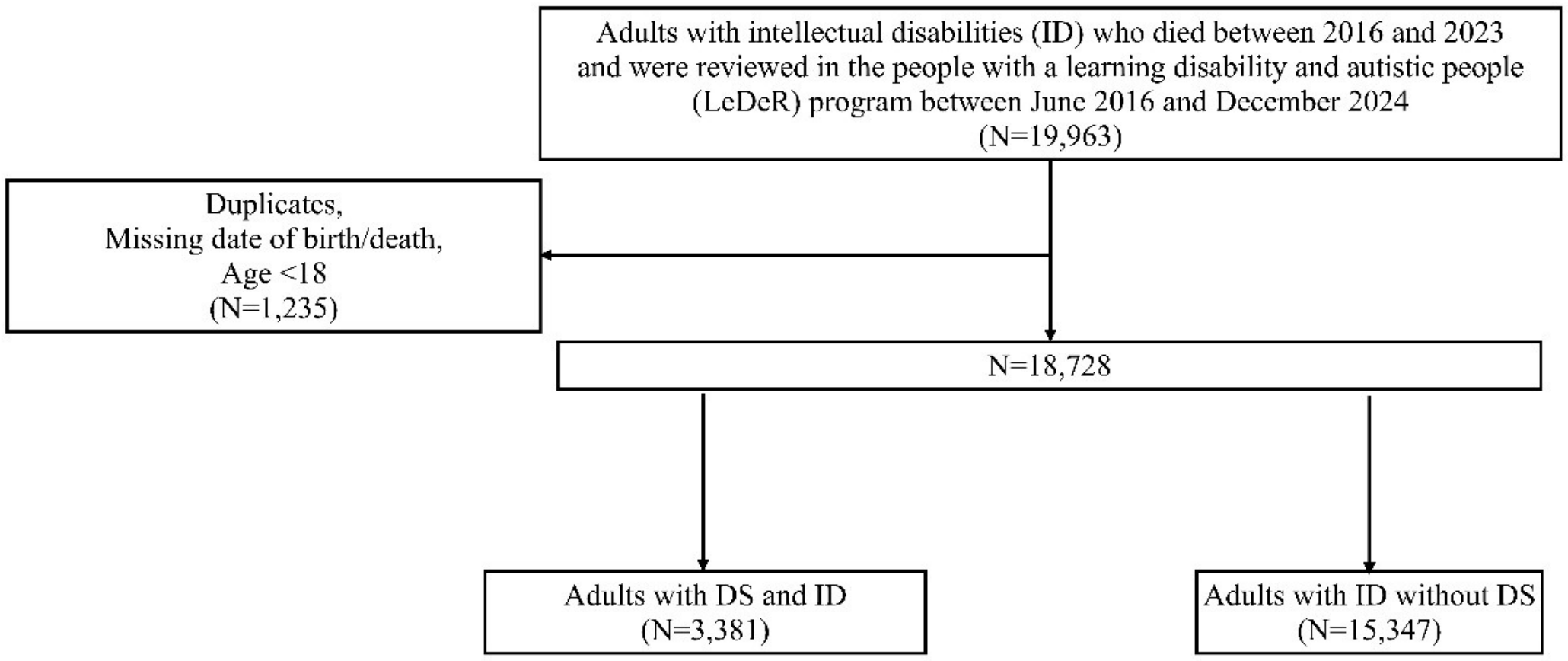

Abbreviations: DS, Down syndrome; ID, intellectual disability.

The Learning from Lives and Deaths – people with a learning disability and autistic people (LeDeR) cohort included 3,381 adults with Down syndrome (DS) and 15,347 other adults with intellectual disabilities (ID).

© 2026 Bokor L et al. *JAMA Network Open*.

eFigure 2. Survival curve by intellectual disability level and Down syndrome status

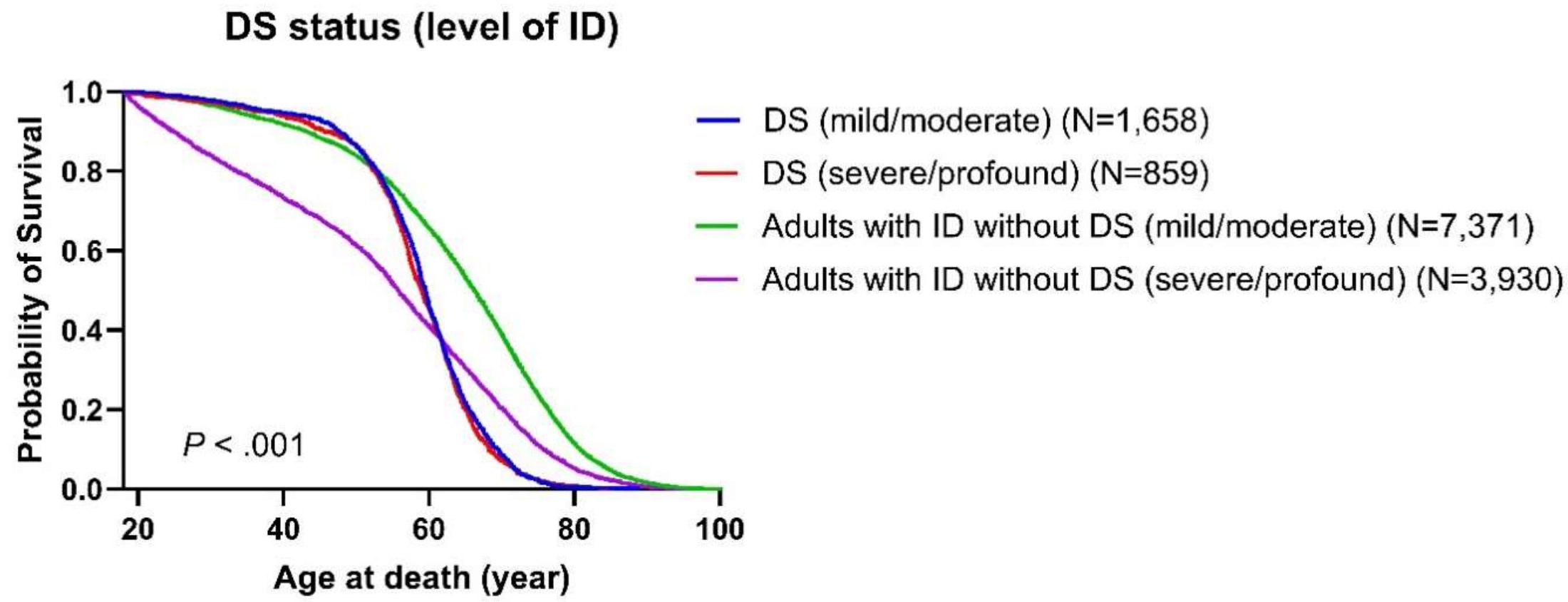

Time point (year)	20	40	60	80	100
DS (mild/moderate)					
At risk	1,658	1,571	772	9	1
Events	0	87	886	1,649	1,657
DS (severe/profound)					
At risk	855	808	393	7	1
Events	4	51	466	852	858
Adults with ID without DS (mild/moderate)					
At risk	7,346	6,774	4,844	841	3
Events	25	597	2,527	6,530	7,368
Adults with ID without DS (severe/profound)					
At risk	3,788	2,885	1,613	207	1
Events	142	1,045	2,317	3,723	3,929

Abbreviations: DS, Down syndrome; ID, intellectual disability.

Among adults with Down syndrome, median age at death did not differ between those with mild to moderate intellectual disability and those with severe to profound intellectual disability (59.5 vs 59.0 years; P = .22). In both Down syndrome subgroups, median age at death was lower than that in adults with mild to moderate intellectual disability without Down syndrome (66.3 years) but higher than that in adults with severe to profound intellectual disability without Down syndrome (55.8 years) (P < .001).

© 2026 Bokor L et al. *JAMA Network Open*.

References:

1. Avoidable mortality: OECD/Eurostat lists of preventable and treatable causes of death (January 2022 version): Organisation for Economic Cooperation and Development; 2022 [Available from: https://www.oecd.org/content/dam/oecd/en/data/datasets/oecd–health–statistics/avoidable–mortality–2019–joint–oecd–eurostat–list–preventable–treatable–causes–of–death.pdf.

2. Coronavirus disease (COVID–19) pandemic: World Health Organization; [Available from: https://www.who.int/europe/emergencies/situations/covid–19.

© 2026 Bokor L et al. *JAMA Network Open*.
