# Supplementary material for: Avoidable Mortality in Adults With Down Syndrome or With Other Intellectual Disabilities
Source: JAMA Netw Open. 2026 Nov 11;9(11):e2643229. doi: 10.1001/jamanetworkopen.2026.43229 (PMC13756131; doi:10.1001/jamanetworkopen.2026.43229)
Supplement: Supplementary file 2 [file jamanetwopen-e2643229-s002.pdf]

Data Sharing Statement

Bokor. Avoidable Mortality in Adults With Down Syndrome or With Other Intellectual Disabilities. *JAMA Netw Open*. Published November 11, 2026.
doi:10.1001/jamanetworkopen.2026.43229

Data
Data available: No

Additional Information
Explanation for why data not available: Individual-level data from the LeDeR mortality review programme cannot be made publicly available because they are potentially identifiable and are held under NHS information governance restrictions and Section 251 approval from the Health Research Authority's Confidentiality Advisory Group (Ref: 20/CAG/0067), which does not permit onward disclosure to third parties. Enquiries regarding access to LeDeR data should be directed to the LeDeR programme team at england.lederprogramme@nhs.net. General population mortality statistics used for comparison were obtained from the UK Office for National Statistics and are publicly available at https://www.nomisweb.co.uk/datasets/mortsa. The aggregated data underlying all tables and figures in this manuscript are provided within the manuscript and its Supporting Information files, with the exception of cells containing fewer than five deaths, which are suppressed in accordance with NHS statistical disclosure control requirements.
